# T Cell Exhaustion and CAR-T Immunotherapy in Hematological Malignancies

**DOI:** 10.1155/2021/6616391

**Published:** 2021-02-25

**Authors:** Lu Tang, Yinqiang Zhang, Yu Hu, Heng Mei

**Affiliations:** ^1^Institute of Hematology, Union Hospital, Tongji Medical College, Huazhong University of Science and Technology, Wuhan, 430022 Hubei, China; ^2^Hubei Clinical Medical Center of Cell Therapy for Neoplastic Disease, Wuhan, 430022 Hubei, China

## Abstract

T cell exhaustion has been recognized to play an immunosuppressive role in malignant diseases. Persistent tumor antigen stimulation, the presence of inhibitory immune cells and cytokines in tumor microenvironment (TME), upregulated expression of inhibitory receptors, changes in T cell-related transcription factors, and metabolic factors can all result in T cell exhaustion. Strategies dedicated to preventing or reversing T cell exhaustion are required to reduce the morbidity from cancer and enhance the effectiveness of adoptive cellular immunotherapy. Here, we summarize the current findings of T cell exhaustion in hematological malignancies and chimeric antigen receptor T (CAR-T) immunotherapy, as well as the value of novel technologies, to inverse such dysfunction. Our emerging understanding of T cell exhaustion may be utilized to develop personalized strategies to restore antitumor immunity.

## 1. Introduction

T cells recognize tumor antigens expressed by cancer cells and induce tumor rejection in vivo [1]. However, multiple negative immunoregulatory pathways impede T cell-mediated tumor destruction in the tumor microenvironment (TME) [2]. In context of persistent antigen stimulation, T cells derived in TME demonstrate the characteristics of exhaustion that leads to a progression towards terminal differentiation [3]. Chimeric antigen receptor T (CAR-T) immunotherapy has been celebrated as a breakthrough due to substantial benefits observed in clinical trials with patients suffering from relapsed or refractory hematological malignancies, such as B cell malignancies and multiple myeloma (MM). Nevertheless, CAR-T cells prepared from dysfunctional T cells may have weakened targeting and effector functions, as well as obstacles in cell proliferation and persistence in vivo, which may explain the high recurrence rate after CAR-T therapy [3]. Herein, we review the hallmarks of exhausted T cells induced in malignant diseases. A better understanding of mechanisms of T cell dysfunction from a fundamental biological perspective will allow optimization for risk stratification and provide novel avenues for the restoration of intratumoral T cell activity.

## 2. Discovery of T Cell Exhaustion

T cell exhaustion, first proposed in a mouse model of lymphocytic choroid meningitis virus (LCMV) infection [4], is a state of T cell dysfunction characterized by the stepwise loss of effector functions during chronic infections and neoplastic disease [5]. Exhausted T cells are constantly stimulated by chronic inflammatory pathogens or tumor antigens and gradually lose their abilities of antigen recognition, proliferation and activation, secretion of interleukin-2 (IL-2) and tumor necrosis factor *α* (TNF-*α*), or completely lose their abilities to produce interferon-*γ* (IFN-*γ*), chemokines, and degranulation, which finally leads to the stepwise loss of effector functions and impaired elimination of viral or tumor antigens [6, 7].

## 3. Characteristics for T Cell Exhaustion

The data of gene expression profiles suggested that the most notable features of exhausted T cells are (1) upregulation or coexpression of inhibitory receptors; (2) major changes in T cell receptor and cytokine signaling pathways; (3) altered expression of genes involved in chemotaxis, adhesion, and migration; (4) expression of key transcription factors; and (5) profound metabolic and bioenergetic deficiencies [7]. Although inhibitory receptors can be temporarily expressed on the surface of activated effector T cells, they will soon be downregulated at a later stage in normal immune states; if these inhibitory receptors cannot be downregulated, they will continue to be highly expressed on the surface of T cells, which is a characteristic change of T cell exhaustion [5, 8].

## 4. Mechanisms of T Cell Exhaustion

### 4.1. Sustained Antigenic Stimulation

**Figure 1:**
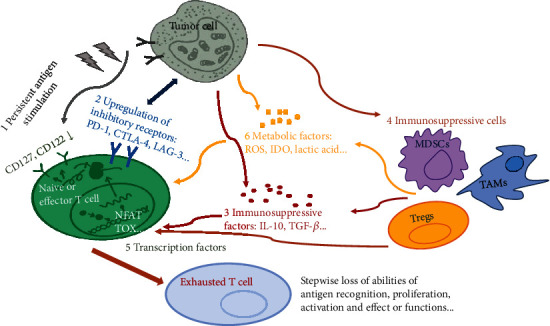
Mechanisms of T cell exhaustion induced in TME.

The important mechanism for the production of virus-specific CD8+ T cells after acute infection is the self-renewal mediated by IL-7 and IL-15 without antigenic stimulation. However, virus-specific CD8+ T cells produced during chronic infection express low levels of CD127 (IL-7 receptor alpha chain) and CD122 (IL-2 and IL-15 receptor beta chains). The subsequent poor response to IL-7 and IL-15 results in long-term maintenance of CD8+ T cells through epitope-specific T cell receptor (TCR) signals but not antigen dependently, which accelerates T cell exhaustion [9]. Therefore, once the process of T cell exhaustion begins, simply removing antigenic stimulation may not necessarily restore the normal proliferation and differentiation of virus-specific CD8+ T cells. If antigenic stimulation is removed earlier or epitope escape occurs, it may be possible for the restoration of some virus-specific CD8+ T cells [9, 10]. A higher level of or more frequent antigenic stimulation usually leads to virus-specific CD8+ T cell exhaustion or lack of immune response cells, while a lower level of antigenic stimulation causes enhanced virus-specific CD8+ T cell response during chronic infection [10]. To sum up, the level and duration of antigenic stimulation may be a key factor in the process of T cell exhaustion (Figure 1).

### 4.2. Inhibitory Receptors

Importantly, exhausted T cells demonstrate upregulation of multiple inhibitory receptors/immune checkpoints that bind to their ligands expressed by tumor cells (Figure 1), antigen-presenting cells (APCs) and myeloid-derived suppressor cells (MDSCs) in TME, such as programmed death 1 (PD-1), cytotoxic T lymphocyte antigen 4 (CTLA-4), lymphocyte activation gene 3 protein (LAG-3), T cell immunoglobulin domain and mucin domain-containing protein 3 (TIM-3), and TIGIT (T cell immunoreceptor with Ig and ITIM domains) [11–14]. PD-1 and its ligand (PD-L1) are the most important inhibitory receptors in T cell exhaustion, and their interaction inhibits downstream signal transduction and the proliferation and cytotoxicity of T cells [11]. When CTLA-4 is combined with the costimulatory ligands CD80 and CD86, it will trigger inhibitory signals of T cells and reduce the production of IL-2 [15, 16]. Moreover, CTLA-4 also inhibits the protein kinase B (PKB, also termed as AKT) signals and then increases the expression of glucose transportase 1, ultimately damaging the glycometabolism and T cell function [15, 16]. LAG-3, a kind of major histocompatibility complex II (MHC-II) ligand, can inhibit T cell proliferation and effector functions when combined with the MHC-II molecules on APCs through the transduction of regulatory T cell- (Treg-) mediated immunosuppressive signals [13]. TIM-3 is a glycoprotein expressed on the surface of T cells and DCs and induces T cell apoptosis when combined with galectin-9 [17]. The role of TIGIT in T cell exhaustion has also been confirmed: it exerts immunosuppressive effects by binding to CD155 with high affinity and interacts with CD112 and other homologous ligands with low affinity [18, 19]. Furthermore, it is generally admitted that the more inhibitory molecules coexisting on the surface of T cells, the more serious the exhaustion of T cells.

### 4.3. Immunosuppressive Factors

Immunosuppressive factors, such as IL-10 and tumor growth factor *β* (TGF-*β*), can be secreted by tumor cells, Tregs, and tumor-associated macrophages (TAM) [20–23]. IL-10 is related to T cell dysfunction during persistent viral infection, and blocking receptors of IL-10 with neutralizing antibodies subside persistent infection symptoms and enhance the secretion of IFN-*γ* of virus-specific CD8+ T cells [20]. IL-10 and IL-35 derived from Tregs directly act on B lymphocyte-induced maturation protein 1 (Blimp-1) to induce the expression of a variety of inhibitory receptors [21, 22]. TGF-*β* is also a key cytokine that contributes to the activation of Treg and tumor angiogenesis [22], which blocks the IFN-mediated expression of MHC-II molecules, inhibit the differentiation and maturation of APCs, inhibit the production of perforin and granzyme, and thereby impair T cell function [24]. More interestingly, IL-2 is identified as an environmental cue to induce CD8+ T cell exhaustion in TME, which is observed in both mouse models and patients with cancer [25]. Continuously, a high level of IL-2 leads to persistent activation of signal transducers and activators of transcription 5 (STAT5) in CD8+ T cells and thereby causing a coordinated upregulation of inhibitory receptors and downregulation of cytokines and effector molecules through the 5-hydroxytryptophan (5-HTP)/aryl hydrocarbon receptor (AhR) nuclear translocation signaling pathways [25].

### 4.4. Immunosuppressive Cells

TME can be defined as the cellular environment in which the tumor exists and generally consists of immunosuppressive cells, such as Tregs, TAMs, and MDSCs, which together cause dysregulated immune response and form a barrier for cellular immunotherapy [8, 23]. Tregs, a type of T cell subgroup with a specific immunophenotype (CD25+ Foxp3+/CD127-), inhibit activated effector T cells through cell-to-cell contact and the secretion of soluble cytokines (such as IL-10, IL-35, and TGF-*β*) to maintain the balance of immune response and play an important role in immune tolerance [6, 26]. Tregs are recruited in hematological malignancies and exert immunosuppressive effects through direct and indirect induction of T cell senescence and exhaustion, but the specific mechanisms have not been clarified [27]. Moreover, the effector function of T cells improved when the Tregs were depleted, indicating that the recruitment of Tregs may explain for disease persistence in leukemia [28]. In TME, TAMs tend to transform to M2 subtype to produce chemokine ligand 22 (CCL22) but not IL-12, thereby promoting the activation of Tregs to inhibit T cell function [29]. MDSCs suppress antigen-specific T cell function by nitrosylation of MHC peptide and TCR complexes, which interrupts T cell target recognition [30]. The recruitment of MDSCs usually occurs during inflammation and cancer and results in the elevation of some immunosuppressive factors, such as indoleamine 2,3 dioxygenase (IDO), arginase 1 (ARG-1), TGF-*β*, and reactive oxygen species (ROS) [30].

### 4.5. Transcription Factors

T cell exhaustion is also associated with altered transcriptional programs and epigenetic regulations. Both Blimp-1 and PD-1 are upregulated in T cells resulted by sustained antigenic stimulation during chronic infection, which seriously impedes normal differentiation and cytotoxic function of T cells [31, 32]. Knocking out of T-bet in CD8+ T cells will promote the expression of PD-1 and then increase the viral load in the body, suggesting that T-bet also has a regulatory effect on T cell exhaustion [33]. Chen et al. discovered that nuclear factor of activated T cell (NFAT) regulated downstream genes that could reduce the response of T cells to tumors [34]. Among them, Nr4a is a transcription factor encoded by one of the downstream genes, and CD8+ T cells from humans with cancer or chronic viral infections expressed high levels of Nr4a transcription factors and displayed enrichment of Nr4a-binding motifs in accessible chromatin regions [34, 35]. Additionally, nuclear factor TOX (thymus high-mobility group box protein) is a crucial regulator of the differentiation of tumor-specific T cells, and TOX-induced exhaustion program serves to prevent the overstimulation of T cells and activation-induced cell death in cancer [36, 37].

### 4.6. Metabolic Factors

It is recognized that T cell differentiation is accompanied by metabolic changes, which can be affected by TME [38]. Excessive consumption of essential metabolites such as glucose and amino acids, elevated production of large amounts of fatty acids and lactic acid, ROS produced by cancer cells, and the hypoxic acidic microenvironment of TME may all minimize the potential of T cells to mediate effector function and drive T cell differentiation to the state of exhaustion [38]. Cancer cells persistently upregulate the transcription of multiple enzymes in the glycolysis pathway (such as hexokinase, phosphofructokinase, and pyruvate kinase) and then produce large amounts of lactic acid to directly inhibit T cells or reduce metabolic adaptability through competitive inhibition [38]. Lactic acid is largely produced by highly glycolytic cancer cells and can suppress the proliferation and cytokine production of human cytotoxic T lymphocytes (CTLs) and reduce cytotoxic activity [39, 40]. Tumor-induced glucose restriction decreases the expression of the metabolic checkpoint EZH2 (enhancer of zeste 2 polycomb repressive complex 2 subunit) in T cells and then limit the polyfunctionality and survival of T cells, indicating that therapeutic manipulation of metabolic checkpoints may represent a promising approach to reverse T cell dysfunction [41]. Cancer cells and MDSCs express IDO, which activates protein kinase 2 (PK2) and inhibit mammalian target of rapamycin (mTOR) signal, resulting in the hindrance of T cell proliferation and survival [38, 42]. Additionally, costimulation domains have been shown to affect the persistence of CAR-T cells through the metabolic characteristics [43]. Inclusion of 4-1BB in the CAR architecture promoted the outgrowth of CD8+ central memory T cells that had significantly enhanced respiratory capacity, increased fatty acid oxidation, and enhanced mitochondrial biogenesis; in contrast, CAR-T cells with CD28 domains yielded effector memory cells with a genetic signature consistent with enhanced glycolysis [43, 44].

## 5. T Cell Exhaustion in Hematological Malignancies

Similar as solid tumors, T cell exhaustion has been observed in various hematological malignancies, such as acute myeloid leukemia (AML), acute lymphoblastic leukemia (ALL), chronic lymphocytic leukemia (CLL), MM, and lymphomas [21, 45–52]. Leukemia-specific T cells in AML patients prone to relapse display exhaustion markers (PD-1+Eomes+T-bet-), whereas absent in patients maintaining long-term CR [45]. A higher proportion of early differentiated memory stem T (T_SCM_) and central memory T (T_CM_) cells from bone marrow demonstrates multiple inhibitory receptors in relapsed patients, and exhausted T cells at relapse display a restricted T cell receptor (TCR) repertoire, impaired effector functions, and leukemia-reactive specificities [45]. TIM-3-mediated interaction between T cells and leukemia cells is reported as a strong risk factor for relapse in pediatric B-lineage ALL, and CD200/TIM-3-signaling is uncovered as a major mechanism of T cell dysfunction [47]. T cells from patients with CLL showed increased expression of exhaustion markers CD244, CD160, and PD-1, as well as the expansion of a PD1+Blimp-1 high subset [48]. Moreover, these abnormal T cells showed functional defects in proliferation and cytotoxicity, with the cytolytic defect caused by impaired granzyme packaging into vesicles and nonpolarized degranulation [48]. Additionally, a subpopulation of exhausted and senescent CD8+ T cells downregulates CD28 and upregulates CD57 and PD-1, characterizing immune impairment and predicting relapse of MM patients after autologous stem cell transplantation (ASCT) [49, 50]. Conclusively, different types of hematological malignancies exhibit various characteristics of T cell exhaustion, but all involve abnormalities of immune cells and relevant cytokines or transcription factors, which provide a new way for reversing T cell exhaustion in immunotherapy.

## 6. T Cell Exhaustion and CAR-T Immunotherapy

At present, CAR-T immunotherapy is mainly autologous; but from the perspective of T cell exhaustion, CAR-T cell therapy has obvious shortcomings. As mentioned before, T cells derived in TME from patients show the characteristics of exhaustion that can lead to a progression towards terminal differentiation [3]. CAR-T cells prepared from functionally impaired T cells may have weakened targeting and effector functions, as well as obstacles in cell proliferation and persistence in vivo [3]. Fraietta et al. reported that CAR-T cells from complete responding patients with CLL were enriched in memory-related genes, including IL-6/STAT3 signatures, whereas T cells from nonresponders upregulated programs involved in effector differentiation, glycolysis, exhaustion, and apoptosis [53]. More importantly, sustained remission was associated with an elevated frequency of CD27+CD45RO-CD8+ T cells before CAR-T cell generation, and these lymphocytes possessed memory-like characteristics [53]. Despite the expansion of CAR-T cells in vivo, relapse commonly occurs after the achievement of CR, and the target antigen may remain on the surface of the tumor cells or not [54–56]. Target-positive relapses occur in patients with loss of functioning CAR-T cells due to the host immunological processes to reject the CAR-T cells [55, 56]. Relapse after CAR-T cell therapy in patients with relapsed/refractory ALL can be roughly divided into CD19-positive recurrence and CD19-negative recurrence [57, 58]. The positive recurrence is manifested as poor proliferation and killing function of CAR-T cells, which may be due to the application of cytotoxic drugs before CAR-T cell infusion, the presence of persistent leukemic blasts in bone marrow and blood, and the defective scFv (single-chain variable fragment) binding kinetics and co-stimulatory molecules [59]. Therefore, the reversal of cancer-related T cell exhaustion is the key point to enhance the therapeutic effects of CAR-T immunotherapy.

## 7. Strategies to Improve CAR-T Cell Exhaustion

### 7.1. Blockers of Inhibitory Receptors

**Figure 2:**
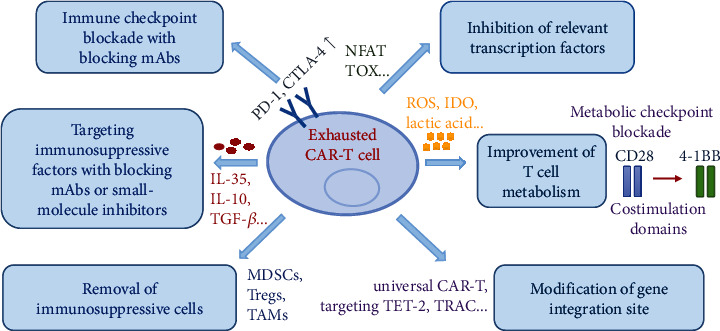
Promising strategies to inverse T cell exhaustion in CAR-T immunotherapy.

Recent studies suggested that the blockade of checkpoint was one of the most widely used and successful strategies to combat T cell exhaustion (Figure 2) [60, 61]. Targeted drugs, such as PD-1, PD-L1, and CTLA-4 antibodies, have been used independently or in combination with CAR-T cell therapy and have been deemed successful in some patients [61]. Strategies to suppress the expression of PD-1 on CAR-T cells include the combination with PD-1/PD-L1 antibodies, modification of CAR-T cells to secrete PD-1/PD-L1 antibodies, or gene modification through knockout of PD-1 [62–65]. CAR-T cells that can secrete PD-L1 or PD-1 antibodies have been designed and demonstrated higher cytotoxicity toward tumors as well as longer persistence in vivo [63, 64]. Ren et al. designed an innovative CAR-T cell through knockout of PD-1, which showed enhanced antitumor effects in vitro and in vivo in mouse prostate cancer models [62, 65]. In addition, other checkpoints have also been knocked out through gene editing technology to improve the anti-tumor activity of CAR-T cells, such as CTLA-4, TIM-3, and LAG-3 [61, 65]. For example, the CRISPR/Cas9 (clustered regularly interspaced short palindromic repeats/caspase 9) system was used to generate a universal CAR with double knockout of PD-1 and CTLA-4, effectively improving the antitumor effects of CAR-T cells [65]. To conclude, these preclinical data prove that the combination with blockade of immune checkpoints is a more reasonable treatment strategy to improve CAR-T cell therapy.

### 7.2. Inhibition of Immunosuppressive Cells

It has been proved that the combination of Treg removal and CAR-T cell therapy could enhance the antitumor effects in animal models of hematological and solid cancers [66]. In addition, Long et al. reported that combined therapy using GD2-CAR T cells plus diminishing the suppressive effects of MDSCs could enhance the efficacy of CAR-T therapy in a xenograft sarcoma model [67]. Moreover, the immunotoxin gemtuzumab ozogamicin can deplete MDSCs, providing a translational approach to reactivate T cell and CAR-T cell responses against multiple cancers [68]. Therefore, inhibition of these immunosuppressive cells proposes a new way to enhance therapeutic efficacy of CAR-T therapy.

### 7.3. Inhibition of Immunosuppressive Factors

The deletion of IL-10 and IL-35 lead to a significant downregulation of exhausted gene and upregulation of memory-related transcription profiles [22]. Lenalidomide has been shown to reverse the T cell exhaustion by inhibiting IL-10-induced STAT3 (signal transducer and activator of transcription 3) phosphorylation in patients with chronic lymphocytic leukemia (CLL) [69]. Tang et al. demonstrated that knocking out the endogenous TGF-*β* receptor II (TGFBR2) in CAR-T cells with CRISPR/Cas9 technology could reduce the induced the conversion of Tregs and prevent the exhaustion of CAR-T cells, and TGFBR2-edited CAR-T cells showed better in vivo tumor elimination efficacy both in cell line-derived xenograft and patient-derived xenograft solid tumor models [70]. In addition, several gene modification strategies have also been developed to enable T cells to resist tumor suppression in TME, such as transgene expression of dominant negative receptors or signal converters, which can transform suppressive signals into stimulating signals [71, 72].

### 7.4. Inhibition of Relevant Transcription Factors

Knocking out of Nr4a family proteins in CAR-T cells showed stronger effector functions and could effectively shrink tumors in a mouse model [34]. In addition, the knocking out of two newly discovered transcription factors TOX and TOX2 could also improve the response of CAR-T cells to melanoma [36]. As TOX and Nr4a transcription factors are critical for the transcriptional program of CD8+ T cell exhaustion downstream of NFAT, disruption of TOX and Nr4a expressions or activities could be promising strategies for cancer immunotherapy [73]. Recent studies also suggested that hematopoietic progenitor kinase 1 (HPK1) was a mediator of T cell dysfunction and an attractive druggable target to improve immune therapy responses [74]. In a word, these preclinical studies are aimed at solving the problem of T cell exhaustion in CAR-T immunotherapy through the regulation of transcription factors.

### 7.5. Improvement of T Cell Metabolism

Increased numbers of memory T cell subsets were observed in the use of inhibition of glycolysis when culturing T cells, as well as enhanced cell proliferation and reduced exhaustion markers, suggesting that glycolysis may be an important factor that affects T cell metabolism [53]. The interaction between PD-1 and PD-L1 can also regulate early glycolysis levels and mitochondrial changes and inhibit the transcriptional coactivator [75, 76]. Metformin can inhibit the function of mitochondria by reducing the consumption of aspartic acid, thereby reducing the oxygen consumption of tumors and reducing the level of hypoxia in TME [77]. Amino acids such as glutamine and arginine are also essential for T cell proliferation and resistance to exhaustion, but both are usually depleted in TME [78, 79]. Inhibition of degradation or supplement of arginine and glutamine has been shown to promote memory CD8+ T cell differentiation and hold back T cell differentiation to the terminal exhaustion stage [79]. The influence of signaling domains of coreceptors CD28 and 4-1BB on the metabolic characteristics of human CAR T cells also provides new avenues for CAR-T therapy [43]. Therefore, metabolic targets can be exploited therapeutically for the development of approaches to increase the efficacy of immunotherapies.

### 7.6. Modification of Gene Integration Site

Gene integration sites affect the activation and proliferation of T cells and thus significantly affect the function of CAR-T cells [80–82]. Fraietta et al. reported a case that 94% of CAR-T cells originated from a single clone in which lentiviral vector-mediated insertion of the CAR transgene disrupted the methylcytosine dioxygenase TET2 (ten-eleven translocation 2) gene at the peak of the response, and TET2-disrupted CAR T cells exhibited an epigenetic profile consistent with altered T cell differentiation and, at the peak of expansion, displayed a central memory phenotype [80]. It has been demonstrated that targeting the CAR to the TRAC (T cell receptor *α* chain) locus averts tonic CAR signaling and establishes effective internalization and reexpression of the CAR following single or repeated exposure to antigen, delaying effector T cell differentiation and exhaustion [81]. Qasim et al. designed a universal CAR19 (UCART19) T cell by lentiviral transduction of nonhuman leukocyte antigen-matched donor cells and simultaneous transcription activator-like effector nuclease- (TALEN-) mediated gene editing of TRAC and CD52 gene loci, which showed higher persistence and antitumor efficacy [82]. Targeted delivery of CAR into the T cell genome is expected to enhance antitumor effects, but further research is needed to determine the feasibility and safety of this technology.

## 8. Concluding Remarks

T cell exhaustion has been recognized to play an immunosuppressive role in both hematological and solid tumors. Understanding the underlying mechanisms of direct and indirect induction of T cell exhaustion by TME will allow optimization for risk stratification and provide new avenues for the restoration and recovery of T cell exhaustion in CAR-T immunotherapies. Specifically, persistent tumor antigen stimulation, the presence of inhibitory immune cells and cytokines in TME, upregulated expression of inhibitory receptors, changes in related transcription factors of T cells, and metabolic factors may be possibilities of T cell exhaustion. As our understanding of the mechanisms driving cancer-induced T cell exhaustion improves based upon preclinical and clinical studies, we expect that innovative combinatorial immunotherapies will emerge to enhance the therapeutic effects of T cell adoptive immunotherapy and improve the clinical outcomes of patients with hematological malignancies.
